# Smartphone Photography in Plastic Surgery: Advancements, Ethics, and Best Practices

**DOI:** 10.1093/asjof/ojaf065

**Published:** 2025-06-19

**Authors:** Abdulla A Fakhro, Abeer Farhan

## Abstract

The extensive integration of smartphone photography has tremendously influenced plastic surgery, providing unmatched convenience, accessibility, and image quality. This comprehensive review explores the various applications of smartphone technology in the care of plastic surgery patients, from preoperative planning to postoperative monitoring. It emphasizes sophisticated smartphone camera capabilities and the potential for artificial intelligence-driven applications. However, using smartphones gives rise to noteworthy ethical considerations, requiring the implementation of solid regulations, adherence to guidelines, and personnel training to guarantee patient privacy and data security. This article is a valuable resource for healthcare professionals seeking to integrate smartphone photography into their practices while navigating the complex ethical landscape of digital imaging in healthcare.

**Level of Evidence:** 5 (Risk)

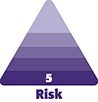

The origins of medical photography can be traced back to the field of plastic surgery, particularly to Gordon Buck, a renowned military plastic surgeon during the Civil War era. He is well-known for being the first physician to incorporate pre- and post-operative photographs into his publications, making a significant contribution to the field. The extensive history of photography highlights its crucial role in patient documentation, analysis of medical conditions, planning surgeries, educating medical professionals, and publishing research findings. High-resolution DSLR cameras have always been conventionally used in clinical photography. However, surgeons have experienced a revolutionary transformation in picture capturing and sharing owing to smartphones. This technology has revolutionized patient care by facilitating rapid communication and decision-making among surgical teams.^1^

Plastic surgeons routinely use smartphones to document and share patient care information. Smartphones have improved productivity in plastic surgery but have also generated concerns about privacy and data security. This review delves into smartphone technology, photography techniques, and ethical guidelines to support plastic surgeons in ethically utilizing smartphones while maintaining patient care standards.

## ENHANCEMENTS IN SMARTPHONE PHOTOGRAPHY

The field of smartphone photography has seen significant transformations with the rapid advancements in mobile technology. Smartphones now rival digital cameras in terms of resolution, software, and functionality, simplifying high-quality photography. Contemporary iPhones and Android phones are equipped with telephoto, wide-angle, and ultra-wide lenses, enabling users to capture close-ups, full-body, and panoramic photographs.^2^

Smartphones equipped with high sensor sensitivity can capture high-quality images in low-light environments, rendering them well-suited for documenting surgical procedures conducted in dimly lit operating rooms. In addition, detachable telephoto lenses allow photographers to magnify subjects without compromising resolution and sharpness.

Smartphones are favored over digital cameras due to their compact design, enabling effortless portability and convenience while on the move. They allow users to easily take and share patient photos for consultation and education, improving patient care and surgical planning.^3^

Numerous surveys consistently demonstrate that smartphone photography is widely used in plastic surgery. A recent study revealed that 89.1% of plastic surgeons and 100% of surgical residents captured patient photographs using smartphones.^4^ A separate study discovered that 99% of those undergoing plastic surgery training use smartphones to capture clinical photographs.^5^ However, these findings also highlight the need for more privacy education and compliance, as many respondents stored patient images alongside personal photos. As smartphone photography becomes more common in healthcare, personnel must be trained on secure image storage and Health Insurance Portability and Accountability Act of 1996 (HIPAA) compliance to protect patient privacy.^4^

### Ethics and Compliance With HIPAA

Confidentiality of patient information is of utmost importance when dealing with clinical photographs. Patient photographs are classified as protected health information. They must be managed in strict accordance with the regulations outlined by HIPAA. This encompasses safeguarding patient confidentiality, ensuring data security, and preserving sensitive content. Patient consent is necessary to utilize clinical photographs for educational or research purposes. However, photos may be exempt if properly de-identified, encrypted, and password-protected, excluding all patient characteristics and information.^6^ Posting clinical photos on widely used messaging platforms such as WhatsApp (Meta Platforms, Inc., Menlo Park, CA), Facebook (Meta Platforms, Inc.), X (X Corp., San Francisco, CA) and Instagram (Meta Platforms, Inc.) gives third parties access to sensitive patient data, compromising privacy. Adhering to compliant methods is essential to mitigate the legal and financial implications that healthcare practitioners may face. Non-compliance may lead to carelessness, invasion of privacy, breaches of fiduciary responsibility, and breaches of confidentiality.^7^

Numerous software firms provide Business Associate Agreements (BAA) to enable hospitals to securely retain patient data within their systems in compliance with HIPAA regulations. Electronic medical records (EMRs) are typically designed to comply with the standards established by HIPAA to ensure the protection of patient privacy. However, EMRs are susceptible to cyberattacks and the unauthorized access and theft of sensitive data. In December 2020, the internationally renowned *Hospital Group* plastic surgery clinic in England experienced a large-scale cyber-attack. Unauthorized individuals illicitly obtained and exfiltrated 900 GB of patient imagery. Subsequently, the perpetrators issued a ransom demand to restate the stolen data.^8^

Clinical photographs should be taken and promptly synchronized with a secure, HIPAA-compliant storage system that allows remote photo deletion in case of physical phone theft. Some examples of secure cloud storage platforms that comply with HIPAA regulations are Box (Box, Inc., Redwood City, CA), Dropbox Business (Dropbox, Inc., San Francisco, CA), and Microsoft Azure (Microsoft Corporation, Redmond, WA). These provide a range of features, including secure file sharing, storage, collaboration, encryption, and access controls. Healthcare enterprises should evaluate the security features, compliance certifications, and willingness to sign a HIPAA-compliant system BAA to guarantee their data’s safety and compliance.^7^

In addition, dual-mode and 2-factor authentication is highly recommended to enhance data security. These identity and access management solutions demand 2 credentials, double identity authentication, and the protection of sensitive data. Canfield’s Mirror Suite (Canfield Scientific, Parsippany, NJ) is a widely used, sophisticated clinical photo storage solution designed specifically for cosmetic practices. Furthermore, the platform’s Photo File technology provides photo editing capabilities and ensures the secure storage of patient photographs in a manner compliant with HIPAA regulations.^8^

Artificial intelligence (AI) is currently being employed to ensure the security of clinical photographs and is under investigation as it progresses. RxPhoto (RxPhoto, Boca Raton, FL) is a mobile solution for medical photography that enables secure capture, management, and sharing of medical images, eliminating the need for costly equipment and ensuring patient data protection. CaptureProof, an AI-powered platform, provides secure storage and transmission of healthcare videos and photos, ensuring HIPAA compliance while enhancing clinical photography efficiency. In the future, medical photography is projected to be transformed by AI in image acquisition, processing, and security.^9^

## EXPERT SMARTPHONE USE IN PLASTIC SURGERY

Current smartphone devices can capture high-definition photographs comparable in quality to those taken by DSLR cameras. Even skilled photographers use their smartphones to take pictures when their specialized equipment is unavailable. However, taking good photographs transcends phone ownership alone. It requires a deep understanding of mobile photography, which can be as complicated as traditional photography.

These principles rely on various operating systems, the most popular being Android OS (Google LLC, Mountain View, CA) and Apple iOS (Apple Inc., Cupertino, CA). The iPhone 15 Pro Max (Apple Inc.) features a sophisticated triple camera system with a 48 MP wide sensor, 12 MP telephoto sensor, and 12 MP ultra-wide sensor, complemented by advanced features like ProRAW and 4K Dolby Vision high dynamic range (HDR) recording. In comparison, leading Android phones such as the Samsung Galaxy S23 Ultra (Samsung Electronics, Suwon, South Korea) and Google Pixel 7 Pro (Google LLC) offer up to 200 MP high-resolution sensors and robust computational photography capabilities, making them strong contenders in the smartphone camera market.^10-12^ These smartphones enable surgeons to quickly capture and share photos, facilitating collaboration and allowing them to monitor patient progress and outcomes from the clinic to the operating room.^13^

Smartphones and mobile photography have significantly improved, proving themselves as valuable tools for transforming clinical images into high-quality medical photos, particularly in plastic surgery. Innovative designs like “tetra prisms” and “periscopes” are used to manipulate the light path within the compact structure of the smartphone device. These techniques, which involve bending the light multiple times before it reaches the sensor, avoid the need for large, elongated lens assemblies usually required by telephoto cameras. They offer a creative solution to the inherent challenges posed by the laws of optics, physics, and engineering in the context of miniaturized devices.^14^

While smartphone photography offers distinct advantages, it also presents challenges, such as varying lighting, composition, and image quality determined by the device and the user's proficiency. Dedicating time to mastering precise smartphone photography techniques is crucial to achieving optimal results. These skills can empower surgeons to elevate patient care, refine their practice, and excel in medical photography.

### Enhancing Smartphone Photography With External Lenses and Accessories

While contemporary smartphones boast sophisticated integrated camera systems, external lenses and accessories can significantly augment their capabilities for clinical photography. These supplementary devices offer enhanced magnification, superior image quality, and specialized functionalities particularly beneficial in medical contexts. Such add-ons enable healthcare professionals to capture high-resolution, detailed images crucial for accurate diagnosis, treatment planning, and medical documentation. The versatility and portability of these accessories, combined with the ubiquity of smartphones, provide a convenient and effective solution for various clinical imaging needs across different medical specialties.

### Macro Lenses

Macro lenses are essential for capturing close-up details of skin conditions, surgical sites, or other small anatomical features. They allow for extreme close-up photography and reveal intricate details that might be missed with standard smartphone cameras. For example, the Easy-Macro lens (Muses Consolidated, LLC, New York, NY), which uses a rubber band mechanism, provides 4× magnification and can be easily attached to most smartphones.

### Telephoto Lenses

Telephoto lenses offer optical zoom capabilities, allowing for detailed images from a greater distance. This can be particularly useful when photographing sensitive areas or when maintaining a sterile field is necessary. For instance, the Moment Tele 58 mm lens (Moment, Inc., Santa Monica, CA) provides 2× optical zoom without sacrificing image quality.

### Wide-Angle Lenses

Wide-angle lenses can be beneficial for capturing broader views of surgical sites or full-body images. These lenses allow clinicians to document a larger area in a single frame, which is helpful for before-and-after comparisons or overall patient documentation.

### Lens Attachment Systems

Various systems exist to attach external lenses to smartphones. Some, like the Moment case system (Moment, Inc.), provide a secure and easy-to-use method for attaching and detaching lenses. Others, like clip-on lenses, offer a more universal fit but may need to be more stable.

### Lighting Accessories

Proper lighting is crucial for clinical photography. External LED or ring lights attached to smartphones can significantly improve image quality, especially in low-light conditions often encountered in clinical settings.

### Image Quality Considerations

External lenses can significantly enhance smartphone photography, but their quality varies. While high-quality lenses from reputable manufacturers minimize distortions and aberrations—especially at the edges—lower-quality options may introduce noticeable image degradation.

In clinical settings, incorporating these external lenses allows clinicians to expand their smartphone’s photographic capabilities. Specialized lenses, such as macro or telephoto attachments, enable the capture of intricate details and improved magnification, enhancing clinical documentation and versatility. Despite advancements in smartphone camera technology, external lenses remain valuable tools for specific imaging needs in healthcare.

### Optical Lens Maintenance

Like DSLR cameras, it is essential to maintain the cleanliness of smartphone lenses to acquire surgical photos of superior quality. Frequent usage of smartphones can lead to smudges and dust accumulating on the lenses, which in turn can reduce the sharpness of images. Periodically, using a pristine microfiber cloth to wipe the lens can be a viable substitute for a lens cleaning kit.^4,15^

### Limitations of Digital Zoom in Surgical Photography

**Figure 1.**
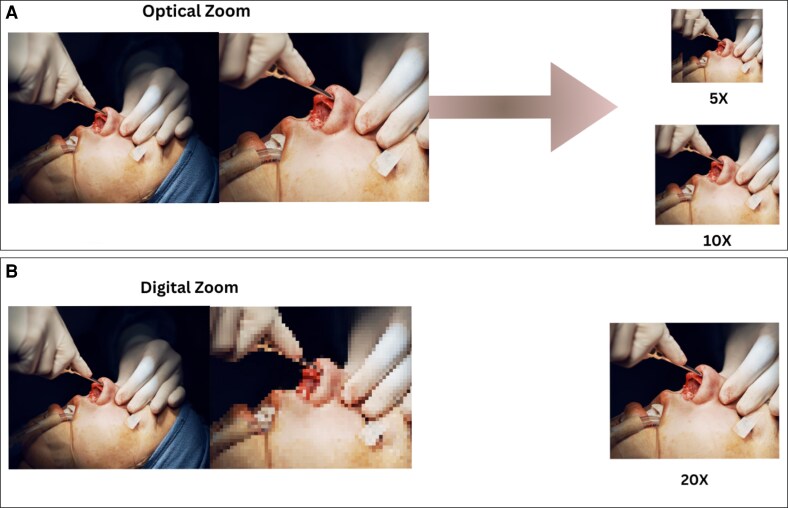
Understanding zoom technologies: optical vs digital zoom. (A) Optical zoom maintains image quality as it is not dependent on resolution, allowing you to zoom in on distant subjects without loss of clarity. (B) Digital zoom relies on resolution, cropping a portion of the image and enlarging it, which can result in a loss of image quality and pixelation.

Excessive digital zoom use is a standard error in smartphone photography, particularly in healthcare. Unlike optical zoom, digital zoom functions by digitally enlarging images, resulting in pixelation and loss of detail. Consequently, this impedes capturing comprehensive pictures of patients’ wounds, surgical sites, and pre- and post-operative photos (Figure 1A, B).

Photographers are recommended to approach their subjects as closely as possible, wherever feasible, to preserve their photographs’ fine details effectively. By doing this, the camera sensor can capture the image at its maximum resolution. Moreover, utilizing macro lens attachments enhances the ability of smartphones to capture intricate close-up shots without relying on digital zoom. These lenses can focus on small details, resulting in precise and meticulous medical images with excellent resolution.^16,17^

Surgeons should be aware of the limitations of their smartphone cameras and use the highest resolution and picture quality options available. Updating the smartphone’s software is recommended, as doing so might improve the camera's performance and add new features. Providing workers with enough training in medical photography best practices can enhance their ability to use their gear, leading to efficient patient documentation.^18^

### Manual vs Auto Focus: Surgical Photography Precision

**Figure 2.**
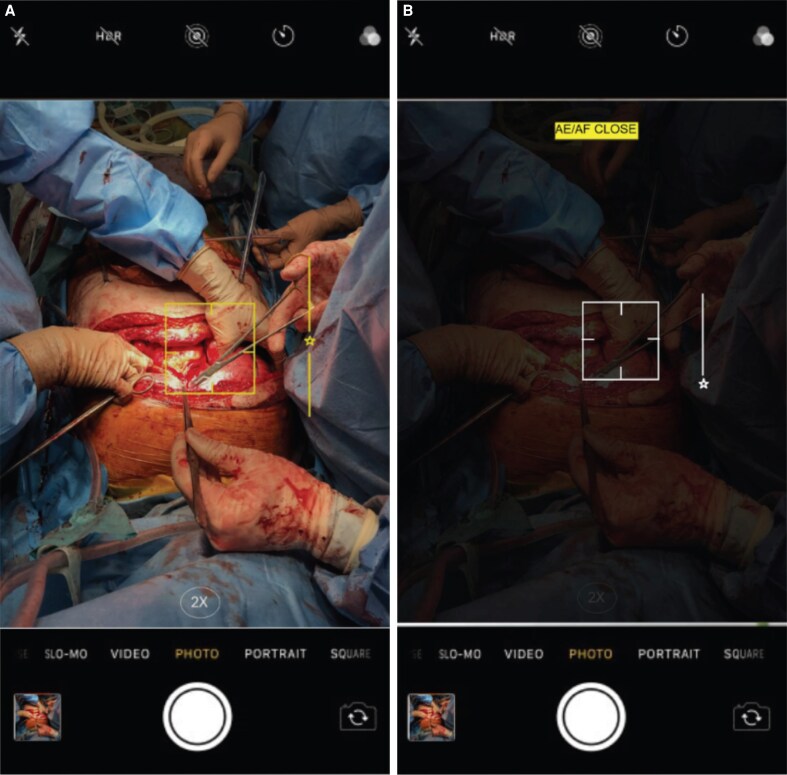
Tap to focus. (A) AE/AF OPEN. (B) AE/AF CLOSED. To adjust focus, tap the square over the subject (in this case operative field). To disable the AE/AF lock, tap and hold on the subject until the AE/AF lock icon appears. Tap the icon again to unlock the settings.

Autofocus is a widely utilized and convenient feature in photography. However, manual focus offers a higher degree of control over image composition. This is particularly crucial in the field of surgical photography. Achieving accurate focus on the operative site is paramount to ensure the clarity and detail required for medical documentation and analysis. Smartphone users can sharpen intricate details and adjust exposure and white balance by tapping and holding the screen to focus. This functionality is invaluable when confronted with complex lighting conditions or situations that require exceptional subject clarity (Figure 2).^19^

### Portrait or Landscape: Choosing the Appropriate Orientation

The orientation of surgical photography, whether portrait or landscape, plays a crucial role in visual communication. Landscape mode is preferred for shooting broad surgical regions or full-body photos. Yet, portrait mode is more suited for capturing vertical compositions, such as facial views or close-ups. Understanding the appropriate utilization of each orientation enables the production of captivating and contextually pertinent photographs.^20^

These guidelines are critical for enhancing smartphone photography and capturing precise, high-quality images. Adherence to these principles guarantees clear and creative documentation of surgical outcomes, extending beyond traditional clinical practices.

### Stabilizing Surgical Photography

When it comes to documenting surgeries using smartphones, it is crucial to have a steady hand to minimize the negative impact of hand tremors on the quality of the images. Smartphones without image stabilization (IS) capabilities might lead to the production of blurry photographs. Modern smartphones are equipped with optical IS (OIS) and electronic IS (EIS), essential technologies that specifically deal with these difficulties.

OIS compensates for hand movements in each frame, which is particularly beneficial in situations with poor lighting. On the other hand, EIS focuses on maintaining stability by assuring a consistent framing. These technical developments significantly improve the clarity and durability of images, increasing surgical documentation's precision.^19^

**Figure 3.**
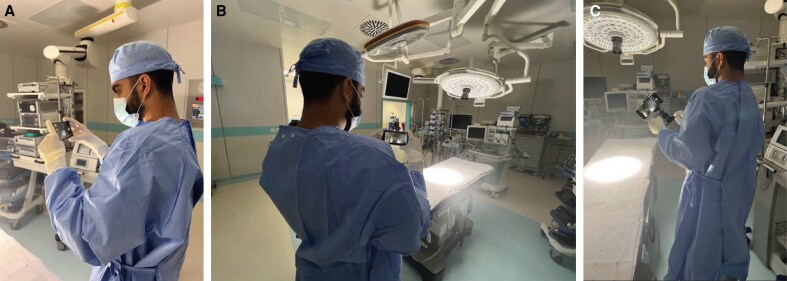
Smartphone utilization in surgical photography: techniques and best practices. (A) A surgical team member holding a smartphone using it to capture photographs in the operating room. (B) The optimal position features the arms close to the body and elbows tucked in while holding a smartphone to achieve stable photography. (C) A specialized surgical photography setup utilizes a tripod for stability, along with a smartphone and lighting to capture high-quality images of surgical procedures.

Ensuring stability in photography is a crucial consideration, regardless of the method employed for stabilization (Figure 3A). When capturing detailed close-up photos for pre- and post-operative documentation, it is imperative to maintain the proximity of the elbows to the body (Figure 3B). This helps to reduce hand movements and improve the clarity of the resulting shots. Tripods can also strategically provide additional stabilization, ensuring accurate and consistent capture of procedural nuances (Figure 3C). The choice of viewpoint dramatically impacts how the subject is portrayed, and paying close attention to even the most minor details becomes crucial. High-angle and profile photos enhance more profound documentation that captures numerous views and clarifies surgical techniques and outcomes.^19^

### Portrait Mode: Enhanced Subject Focus and Visual Aesthetics

Portrait mode has garnered widespread popularity on social media platforms due to its capability to accentuate subjects against defocused backgrounds. This technique has also been applicable in certain popular plastic surgery web pages, where it is used to create DSLR-like “bokeh” images that emphasize the subject while deliberately blurring the backdrop. Modern smartphones now have multiple cameras, including telephoto and wide-angle lenses, facilitating depth measurement and AI algorithms to achieve this visual effect.^21^

While portrait mode holds utility in plastic surgery photography, prudent caution is advised when editing clinical images. Several smartphone and camera software applications enable the application of filters or adjustments to background effects post-capture; yet, these alterations are generally discouraged for plastic surgery documentation and photography. The application of filters and effects to clinical photographs has the potential to distort reality and compromise the accuracy of medical documentation.^21^

Accurate visual depictions of surgical outcomes are critical to the field of surgery. Although minor adjustments to brightness and clarity may be necessary, excessive editing of an image can result in inaccurate perceptions of the outcome. This moral problem is crucial given how much photographic evidence is used in patient education, surgery planning, and result assessment. Ensuring the genuineness of images is essential for upholding established professional norms and fostering confidence between patients and physicians. Significant alterations must be implemented with caution and openness to maintain the validity of surgical records.^9^

### Macro Photography in Plastic Surgery Documentation

The advancement of smartphone cameras has enabled plastic surgery documentation to employ macro photography, a technique previously limited to specialized equipment. Comprehensive surgical site images are vital for planning before surgery, documenting throughout the operation, and monitoring after the procedure. Close-ups at high magnification expose intricate textures and subtle details that are not readily perceptible to the naked eye.

**Figure 4.**
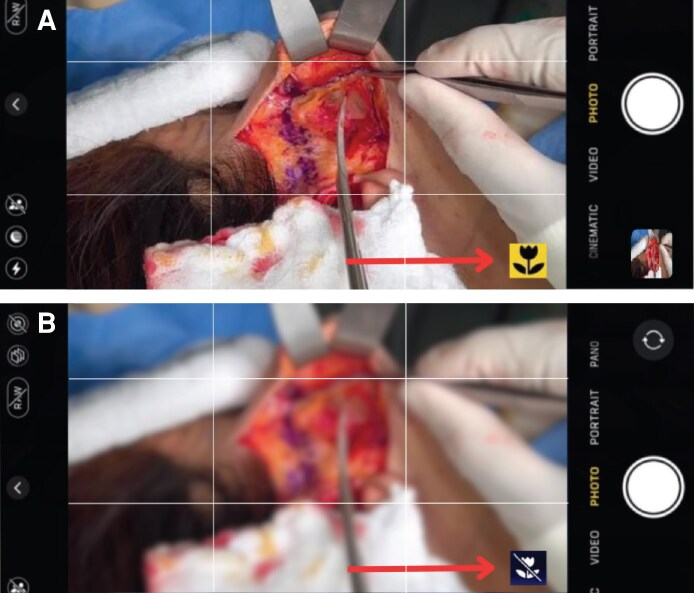
Macro mode activation indicator A flower-shaped icon appears in the viewfinder. (A) When illuminated in yellow, it indicates that macro mode is activated; (B) when not illuminated, the camera is in its standard mode for regular photography. A close-up of the field highlights the distinction between macro and standard modes.

Smartphone cameras with macro capabilities allow for accurate clinical visual documentation. Several smartphone cameras currently provide telescopic lenses and macro settings, enabling users to enlarge small objects and capture intricate details (fig turning on macro). In conventional photography, the lens captures an expansive scene or subject and reduces it to fit within the sensor's dimensions. Contrarily, in macro photography, there is no diminution of the subject's size on the sensor due to the proximity to the subject. By using a firm grip and supplementary external macro lens attachments, surgeons can enhance the caliber and accuracy of their photos, thereby increasing the accessibility and use of macro photography. These technological improvements allow them to use smartphones to capture high-resolution, detailed images of surgery sites and procedures (Figure 4).^22^

For smartphones without built-in macro capabilities, users can imitate macro effects using third-party apps such as Macro by Camera + (LateNiteSoft S.L., Barcelona, Spain) and Manual Camera by Geeky Devs Studio (Geeky Devs Studio, Dhaka, Bangladesh) on Android OS. These apps use software to enable the capture of close-up photos, demonstrating the versatility of smartphone cameras.^4^

### Night Mode Improves Plastic Surgery Photography in Low Light

**Figure 5.**
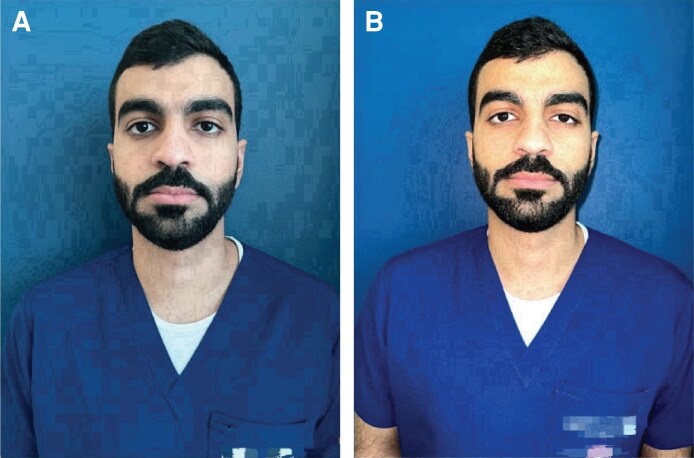
Enhanced low-light photography: night mode and extended shutter speed night mode extends the shutter speed to allow more light to be captured, resulting in brighter and more detailed photographs. (A) Night mode autoflash off. (B) Night mode autoflash on.

It is often challenging to capture high-quality images in low-light circumstances, such as operating rooms and poorly lit clinics. Utilizing the LED flash has the potential to alter the appearance of colors and textures, causing distortion. Night Mode increases the duration of the shutter speed to capture a more significant amount of light, leading to brighter and more intricate photographs. This option improves visibility and accentuates fine details in environments with limited lighting. Nevertheless, it may not be appropriate for capturing moving subjects due to the possibility of motion blur. Choosing the proper mode based on the subject and lighting is essential for optimal image quality (Figure 5).^23^

Maintaining camera steadiness when utilizing night mode is imperative to prevent image blurring caused by prolonged exposures. Even minor movements during image capture can compromise clarity, as the camera's shutter remains open longer to offset low-light conditions. A tripod is recommended for achieving stable and precise long exposures. When a tripod is unavailable, placing the phone on a steady surface or improvising utilizing a malleable gooseneck long arm may be a cost-effective alternative.^24^

### Smartphone Photography Exposure Improves Plastic Surgery Precision

**Figure 6.**
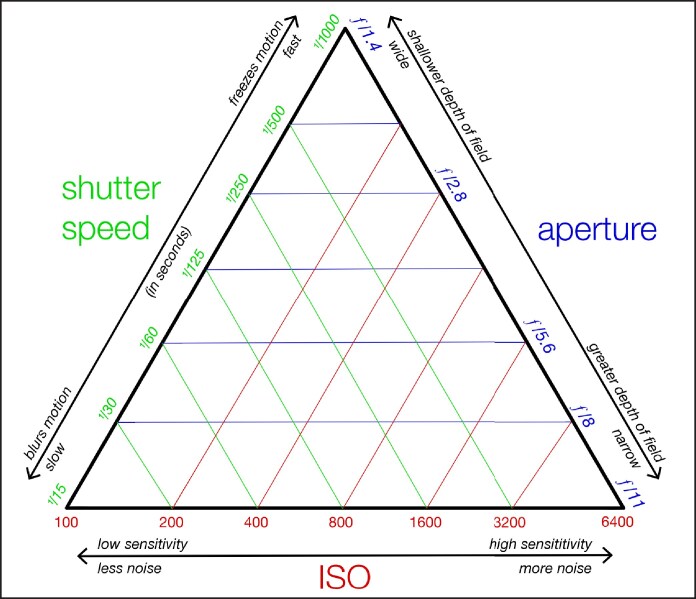
The exposure triangle—aperture, shutter speed, and ISO. Aperture controls the depth of field and light entry, shutter speed determines exposure time and motion capture, and ISO adjusts light sensitivity, balancing image clarity with noise. This figure originally appeared within WClarke and Samsara (https://commons.wikimedia.org/wiki/File:Exposure_triangle_-_aperture, _shutter_speed_and_ISO.svg) under a Creative Commons Attribution-Share Alike 4.0 International license, which permits reproduction of the image with proper attribution to the original work.

Exposure is crucial in establishing precise surgical recordings since it determines the brightness and darkness of an image. Camera lenses manipulate aperture, shutter speed, and ISO to create the Exposure Triangle, which governs the amount of light captured to achieve a desired image. The shutter speed controls the length of time that the camera's sensor is exposed to sunlight, which affects the sharpness of the captured motion. ISO is crucial in regulating image brightness by measuring the sensor's light sensitivity in different lighting conditions (Figure 6).^16^

Smartphone cameras have fixed, non-adjustable apertures, but they can regulate the light they capture. Phones streamline exposure settings with sliders or on-screen controls, allowing users to modify them for optimal image clarity and detail.

**Figure 7.**
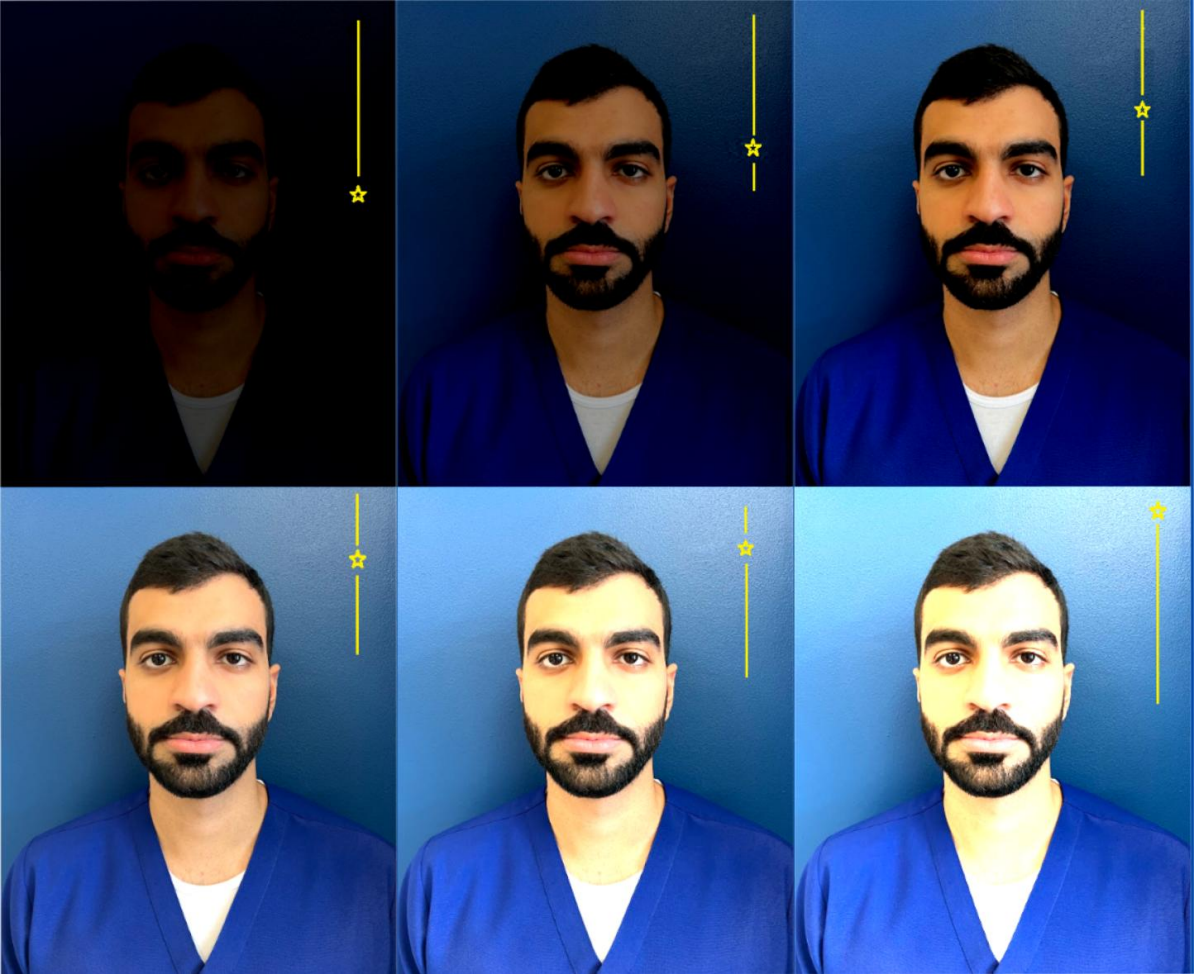
Exposure comparison: illustrating overexposure, underexposure, and ideal exposure. The bottom right corner illustrates a photograph with extreme overexposure, resulting in an excessively bright image. In contrast, the top left corner depicts extreme underexposure, where the image appears overly dark. The top right square represents the ideal exposure in this context.

Understanding exposure helps minimize underexposure, where dark regions lack detail, and overexposure when bright areas lose clarity (Figure 7).

Familiarity with the exposure slider helps photographers adjust brightness accordingly. Reducing exposure can preserve details in bright surroundings, while increasing exposure can improve image visibility without compromising clarity in low-light conditions. Proper exposure control ensures an accurate representation of operative outcomes and patient conditions in plastic surgery documentation.

### Adjusting Exposure to Popular Smartphone Models

Adjusting exposure settings on popular smartphone models is crucial for capturing high-quality clinical photographs in plastic surgery. For iPhone users, tapping on the desired focus area brings up a yellow box with a sun icon. Swiping up or down on this icon adjusts the exposure, with upward swipes increasing brightness and downward swipes decreasing it. More precise control can be achieved by tapping the “^” icon at the top of the screen and using the Exposure slider.

Samsung Galaxy users can tap the screen to focus, which reveals a slider next to the focus circle. Dragging this slider up or down adjusts exposure. For advanced control, switching to Pro mode provides a dedicated exposure compensation (EV) slider. Google Pixel users can tap to focus and then slide the sun icon next to the focus point to adjust exposure.

To achieve proper exposure in clinical photography, consider using your smartphone's histogram feature (if available) to check that the exposure is balanced across the entire image. In challenging lighting conditions, turning on HDR mode can help even out bright and dark areas, resulting in a more uniformly exposed photo. Incorporating a gray card or color checker into your setup can also ensure consistent exposure and accurate colors in your images. Generally, it's better to slightly underexpose photos rather than overexpose them, as this approach helps preserve detail in the highlight areas.

Smartphone photographers can effectively manipulate exposure settings to produce crisp, detailed photographs that meet medical documentation standards, improving the quality of clinical visual records. This option is invaluable when recording tiny changes at the surgery site or capturing skin tones and textures.^25^

### High Dynamic Range (HDR) Photography Enhances Plastic Surgery Documentation

**Figure 8.**
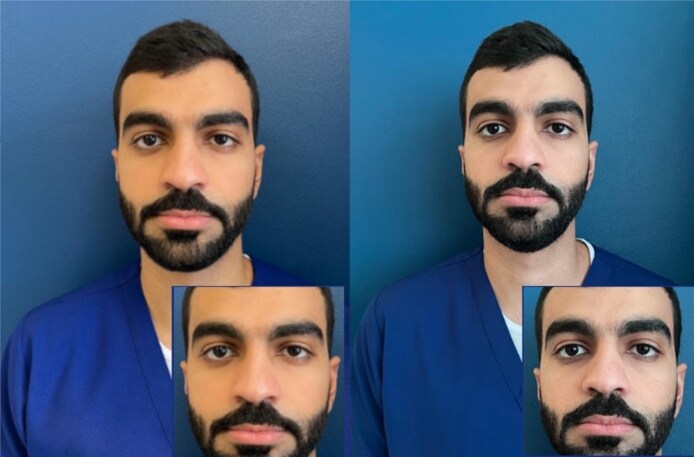
Comparison of HDR+ and standard mode: image quality differences. The image on the left was captured with HDR+ turned off, while the image on the right was taken with HDR+ enabled. The HDR+ photo is noticeably brighter, cleaner, and sharper, revealing significantly more detail in the subject's hair and eyelashes. HDR, high dynamic range.

Mobile devices with HDR photography capabilities offer superior documentation of plastic surgery procedures and outcomes. One must comprehend the concept of dynamic range to achieve clarity in a photograph's dark and brightly illuminated areas. Dynamic range is the ratio between an image's darkest and lightest tones. HDR combines 3 exposures to enhance the overall quality and create a single image. The optimal components from each instance of capturing an image are combined to provide a photograph with enhanced intricacy and evenly distributed illumination. This technique is particularly advantageous for photographs captured at surgical sites, where lighting conditions fluctuate significantly (Figure 8).^3^

HDR mode captures multiple photos, which might slow processing and cause motion blur if the camera is unsteady. In some instances, photographers can select the highest quality photograph using smartphones that offer HDR and non-HDR versions of the same picture. HDR technology may effectively equalize the lighting in portrait photography, resulting in more aesthetically pleasing and intricately captured images of individuals. However, it is advisable to avoid using HDR mode for vividly colorful photographs or high-contrast silhouettes since it can potentially dilute colors or introduce visual distortions due to blending multiple shots.

### Artificial Intelligence (AI) in Clinical Photography for Plastic Surgery

Integrating AI in plastic surgery has opened up new possibilities for enhancing patient care, improving surgical outcomes, and streamlining clinical workflows. AI-powered decision support systems are revolutionizing the field by leveraging machine learning algorithms to analyze vast datasets and provide valuable insights for clinical decision-making. These systems offer enhanced precision and customization in treatment plans, ultimately improving patient outcomes and satisfaction.^26^

In cosmetic surgery, where precision and patient satisfaction are paramount, AI has proven to be a game-changer. By offering highly accurate recommendations, AI allows tailored treatments based on individual patient profiles and predicted outcomes. This technology streamlines routine tasks, enabling surgeons to focus on complex decision-making, ultimately improving efficiency and patient satisfaction. This technology streamlines routine tasks, allowing the surgeons to focus on complex decision-making, ultimately improving efficiency and patient satisfaction. AI supports surgeons in various aspects of their practice. In pre-operative planning, AI can analyze patient anatomy and previous surgical outcomes to suggest the best surgical approach.^27^

AI's real-world applications in cosmetic surgery are already making an impact. Virtual consultations powered by AI platforms can analyze patient photos and provide initial recommendations. Advanced imaging tools can create 3D models of patient anatomy, helping surgeons plan more effectively. Additionally, predictive analytics can forecast trends in patient preferences, allowing practices to stay ahead of the curve.

As the field of plastic surgery continues to evolve, the integration of AI technologies promises to enhance surgical precision, improve patient outcomes, and revolutionize how procedures are planned and executed. However, surgeons must remain aware of AI's benefits and limitations, ensuring its responsible integration into their practice.^9^

### Practical Application of Smartphone Photography in a Medical Setting

Modern plastic surgery increasingly utilizes smartphones for preoperative planning, intraoperative assistance, and postoperative monitoring. High-quality photos can be captured and shared instantly, enhancing visual communication among healthcare practitioners and improving patient consultations and treatment outcomes. Clinical professionals must be well trained in smartphone medical photography to ensure technical proficiency. They must also grasp the legal and ethical consequences of using personal devices to protect patient privacy and data. Smartphones aid plastic surgery decisions, track progress, and improve outcomes.^28^

## FUTURE DIRECTIONS OF SMARTPHONES IN PLASTIC SURGERY

The potential for plastic surgery applications will increase with advancements in smartphone camera quality and image processing techniques. Integrating AI and machine learning into smartphone applications can transform the field of real-time clinical picture analysis and diagnosis.^29^

As technology progresses, ethical considerations regarding data protection and patient confidentiality will change, requiring the establishment of new guidelines and laws to address developing concerns. Smartphones with more advanced features will be used more frequently for remote diagnostics and patient care in plastic surgery.

Smartphones have significantly transformed medical photography in plastic surgery, enhancing the process of documenting and providing care to patients. Clinicians can use smartphones with enhanced resolution, improved low-light capabilities, and detachable lenses to capture comprehensive, high-quality images for diagnosis and treatment planning. The increasing utilization of smartphones in therapeutic settings raises ethical considerations about patient confidentiality and data protection.^30^

Healthcare systems must prioritize training clinical staff to guarantee smartphone photography's responsible and secure use in plastic surgery. This training should encompass instruction on photographic skills and an exploration of the legal and ethical ramifications associated with using personal devices in medicine. Comprehensive policies and procedures are essential to tackle the complexities of digital data security and safeguard patient privacy.^4^

Further research is needed to explore the effects of smartphone photography on patient outcomes and how AI and machine learning might improve image analysis and diagnosis. Studies on HIPAA-compliant systems and cloud storage solutions could benefit healthcare firms seeking to improve their data management practices.

As plastic surgery continues to advance technologically, it is crucial to strike a balance between ethics and innovation. Through continual education, policy development, and privacy compliance, healthcare practitioners can harness the power of smartphone photography to improve patient care while safeguarding sensitive medical information.^31^
